# Identification of hydrogen bonding network for proton transfer at the quinol oxidation site of *Rhodobacter capsulatus* cytochrome *bc*_1_

**DOI:** 10.1016/j.jbc.2023.105249

**Published:** 2023-09-14

**Authors:** Arkadiusz Borek, Anna Wójcik-Augustyn, Patryk Kuleta, Robert Ekiert, Artur Osyczka

**Affiliations:** Department of Molecular Biophysics, Faculty of Biochemistry, Biophysics and Biotechnology, Jagiellonian University, Kraków, Poland

**Keywords:** cytochrome *bc*_1_, proton transfer, enzymatic activity, quinol oxidation, quantum mechanical calculations, *Rhodobacter capsulatus*

## Abstract

Cytochrome *bc*_1_ catalyzes electron transfer from quinol (QH_2_) to cytochrome *c* in reactions coupled to proton translocation across the energy-conserving membrane. Energetic efficiency of the catalytic cycle is secured by a two-electron and two-proton bifurcation reaction leading to oxidation of QH_2_ and reduction of the Rieske cluster and heme *b*_L_. The proton paths associated with this reaction remain elusive. Here, we used site-directed mutagenesis and quantum mechanical calculations to analyze the contribution of protonable side chains located at the heme *b*_L_ side of the QH_2_ oxidation site in *Rhodobacter capsulatus* cytochrome *bc*_1_. We observe that the proton path is effectively switched off when H276 and E295 are simultaneously mutated to the nonprotonable residues in the H276F/E295V double mutant. The two single mutants, H276F or E295V, are less efficient but still transfer protons at functionally relevant rates. Natural selection exposed two single mutations, N279S and M154T, that restored the functional proton transfers in H276F/E295V. Quantum mechanical calculations indicated that H276F/E295V traps the side chain of Y147 in a position distant from QH_2_, whereas either N279S or M154T induce local changes releasing Y147 from that position. This shortens the distance between the protonable groups of Y147 and D278 and/or increases mobility of the Y147 side chain, which makes Y147 efficient in transferring protons from QH_2_ toward D278 in H276F/E295V. Overall, our study identified an extended hydrogen bonding network, build up by E295, H276, D278, and Y147, involved in efficient proton removal from QH_2_ at the heme *b*_L_ side of QH_2_ oxidation site.

Cytochrome *bc*_1_ is one of the membranous energy-converting enzymes of cellular electron transport chains. It operates according to the Q cycle (1, 2, 3) and catalyzes ubiquinol-dependent cytochrome *c* reduction (reviewed in Refs. (4, 5)). The Q cycle contributes to building-up of the proton motive force, which is utilized for ATP synthesis. A key reaction of this cycle that secures its energetic efficiency is the two-electron oxidation of ubiquinol (named hereafter as quinol or QH_2_) taking place at the catalytic Q_o_ (QH_2_ oxidation) site. In this reaction, electrons from QH_2_ reduce the Rieske cluster and heme *b*_L_ and are subsequently transferred through two different cofactor chains: the high-potential c-chain and the low-potential b-chain. Electrons transferred through the b-chain (heme *b*_L_ and heme *b*_H_) reduce ubiquinone (Q) at the Q_i_ site in two sequential steps. In the c-chain (Rieske cluster and heme *c*_1_), electron transfer is possible because of the movement of the head domain of the Rieske iron–sulfur protein (6, 7, 8, 9, 10, 11). Electrons are then transferred from heme *c*_1_ to reduce water-soluble protein partner of cytochrome *bc*_1_–cytochrome *c* (Fig. 1, *A* and *C*).

**Figure 1:**
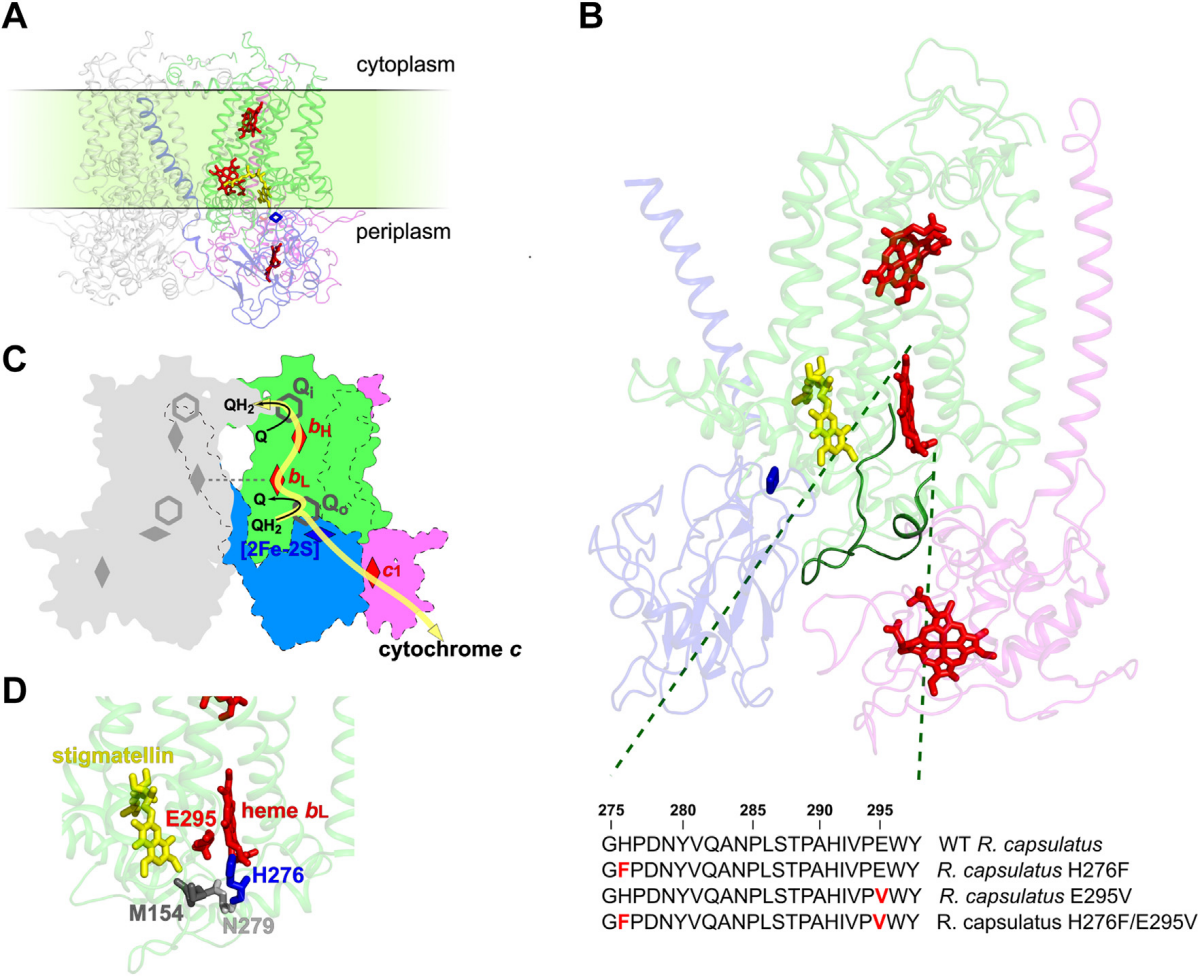
**Overview of the cytochrome *bc*_1_ structure with selected details on the Q_o_ site highlighting the mutated positions.***A*, Crystal structure of dimeric cytochrome *bc*_1_ from *Rhodobacter capsulatus* (Protein Data Bank code: 1ZRT). Subunits of one monomer are colored as follows: cytochrome *b*, *light green*; ISP, *blue*; and cytochrome *c*_1_, *magenta*. Subunits of the second monomer are in *light gray*. *Red* and *dark blue sticks* indicate hemes and Rieske cluster, respectively. *Yellow sticks* represent the stigmatellin molecule. *B*, crystal structure of monomeric cytochrome *bc*_1_ from *R. capsulatus* (structure from Fig. 1*A* rotated by 90°) with highlighted *ef*-loop (*dark green*). The amino acid sequence of this loop (positions 275–297) highlights in *red* the introduced point mutations. *C*, schematic representation of dimeric *R. capsulatus* cytochrome *bc*_1_. Subunits are colored as in Figure 1*A*; *red rhombuses* indicate hemes; *dark gray hexagons*—the Q_o_ and Q_i_ catalytic sites; *yellow arrows* represent the paths of electron transfer within the monomer. For simplicity, the subunit composition and the details of Q cycle reactions are shown only for one of the monomers. The scheme does not consider stoichiometry of the Q cycle. *D*, close-up view of the part of cytochrome *b* showing spatial distribution of mutated positions. ISP, iron-sulfur protein; Q_o,_ quinol oxidation

The deprotonation of QH_2_ upon its oxidation to Q at the Q_o_ site leads to release of protons to the intermembrane space of mitochondria (the periplasm of bacteria). However, the exact paths for the proton transfer from this site to the exterior of the protein remain elusive. It is commonly assumed that one of the protons is transferred to one of the histidine residues coordinating the Rieske cluster (H156 in the purple photosynthetic bacterium *Rhodobacter capsulatus*), whereas another proton is transferred to glutamic acid located in the highly conservative PEWY motif of cytochrome *b* (E295 in *R. capsulatus*). Those two residues are inferred in proton transfer, as in the crystal structures of cytochrome *bc*_1_, they form hydrogen bonds with stigmatellin (7, 12, 13, 14) (Fig. 1, *B* and *D*), a potent inhibitor of the Q_o_ site. Furthermore, a similar position of the natural substrate is inferred from the recent cryo-EM structure of cytochrome *bc*_1_ (15).

However, until now, apart from the observation that histidine is the redox-linked protonation site of the Rieske cluster (16), there is no direct evidence for any of the proton reactions at the Q_o_ site. Mutating the histidine is not informative in this case as it results in disrupting the whole iron–sulfur protein subunit (17). On the other hand, E295 (or its equivalent in yeast) can be replaced by a nonprotonable amino acid, and the enzyme remains active and functional *in vivo* (18, 19, 20, 21). In addition, a comparison of the cytochrome *b* amino acid sequence among different species showed the existence of organisms that do not have a protonable residue in PEWY motif (22). This all indicates that, even if E295 directly participates in proton transfer, alternative routes not involving this residue are also possible. In this context, an interesting concept proposes that a sequence variation within this conserved motif and consequent variations in proton transfer reactions represent adaptation to specific environmental and/or substrate conditions (22).

Of note, the alternative routes might engage water molecules in addition to the protonable amino acid side chains. Indication for that came from molecular dynamics simulations, which imply the possibility that binding at the Q_o_ site involves a coordinated water arrangement in which the water molecules are positioned suitably in relation to the hydroxyls of the QH_2_ ring to act as the primary acceptors of protons (23).

In view of the extensive mutational work done so far on the Q_o_ site, the proton transfer, like the electron transfer, appears relatively tolerant to a single mutational change. In this work, we embarked on this notion and aimed at identifying a combination of mutations that effectively impedes the proton transfer at the heme *b*_L_ side (the region in the vicinity of the PEWY motif) resulting in an enzyme that is nonfunctional *in vivo*. Further identification of the specific second-site compensatory effects complemented with quantum mechanical (QM) calculations allowed us to explain how the extended hydrogen bond network contributes to efficient proton transfer in this region.

## Results and discussion

### Switching off the proton channel requires simultaneous removal of two protonable side chains

The position of the side chain of E295, as seen in the structure of cytochrome *bc*_1_, makes this residue a strong candidate for an immediate acceptor of one of the protons from QH_2_ upon its oxidation at the Q_o_ site (12, 13, 14). On the other hand, in molecular dynamics simulations, E295 tends to rotate away from the bound QH_2_, giving space for a water molecule that can act as a proton acceptor (23). Moreover, mutations of this residue showed that the protonable side chain at this position is not essential for maintaining functionality of the enzyme *in vivo* (19, 20, 21). This implicated that even if E295 was an immediate proton acceptor from QH_2_, alternative routes for proton transfer might exist in this region. We thus reckoned that it would be informative to find a single mutation or a combination of mutations that effectively switches off the proton transfer from QH_2_ making the enzyme nonfunctional *in vivo*.

In search for such mutations, we first targeted H276, a protonable residue in close contact with E295 (Fig. 1*D*). Moreover, H276 is a part of the *ef* loop involved in the binding of zinc (18, 24) believed to be an ion that binds to the proton pathway. We substituted H276 with phenylalanine (H276F) and found that the mutated enzyme was still functional *in vivo* (the mutant cells were able to grow under photosynthetic conditions, Fig. S1). Then we tested enzymatic activities over the pH range from 5.5 to 8.5 and single flash-induced kinetics of electron transfer in membrane chromatophores at pH 7. We compared H276F with WT and E295V. E295V was one of the variants used in the earlier mutational studies on E295 in *R. capsulatus* (18, 19).

**Figure 2:**
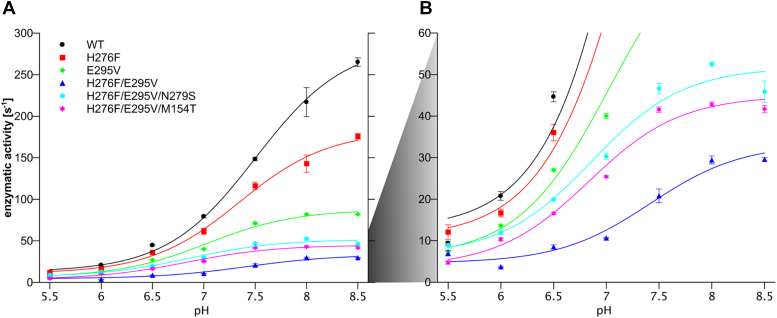
**Enzymatic activities of wild type cytochrome*****bc***_**1**_**and mutants of protonable amino acids in the Q_o_ site region.***A*, pH dependence of the enzymatic activities of various forms of cytochrome *bc*_1_ (WT—*black circles*, H276F—*red squares*, E295V—*green diamonds*, H276F/E295V—*blue triangles*, H276F/E295V/N279S—*cyan pentagons*, and H276F/E295V/M154T—*magenta stars*). *Solid lines* represent the results of fitting an empirically derived equation (Equation 1) to the data points. Error bars represent the standard deviation of the mean of three to five measurements. *B*, close-up of the enzymatic activity graph in the range from 0 to 60 s^−1^ to highlight the effect of reversions (triple mutants: H276F/E295V/N279S and H276F/E295V/M154T) in comparison to original mutation (double mutant H276F/E295V) at lower pH values.

**Figure 3:**
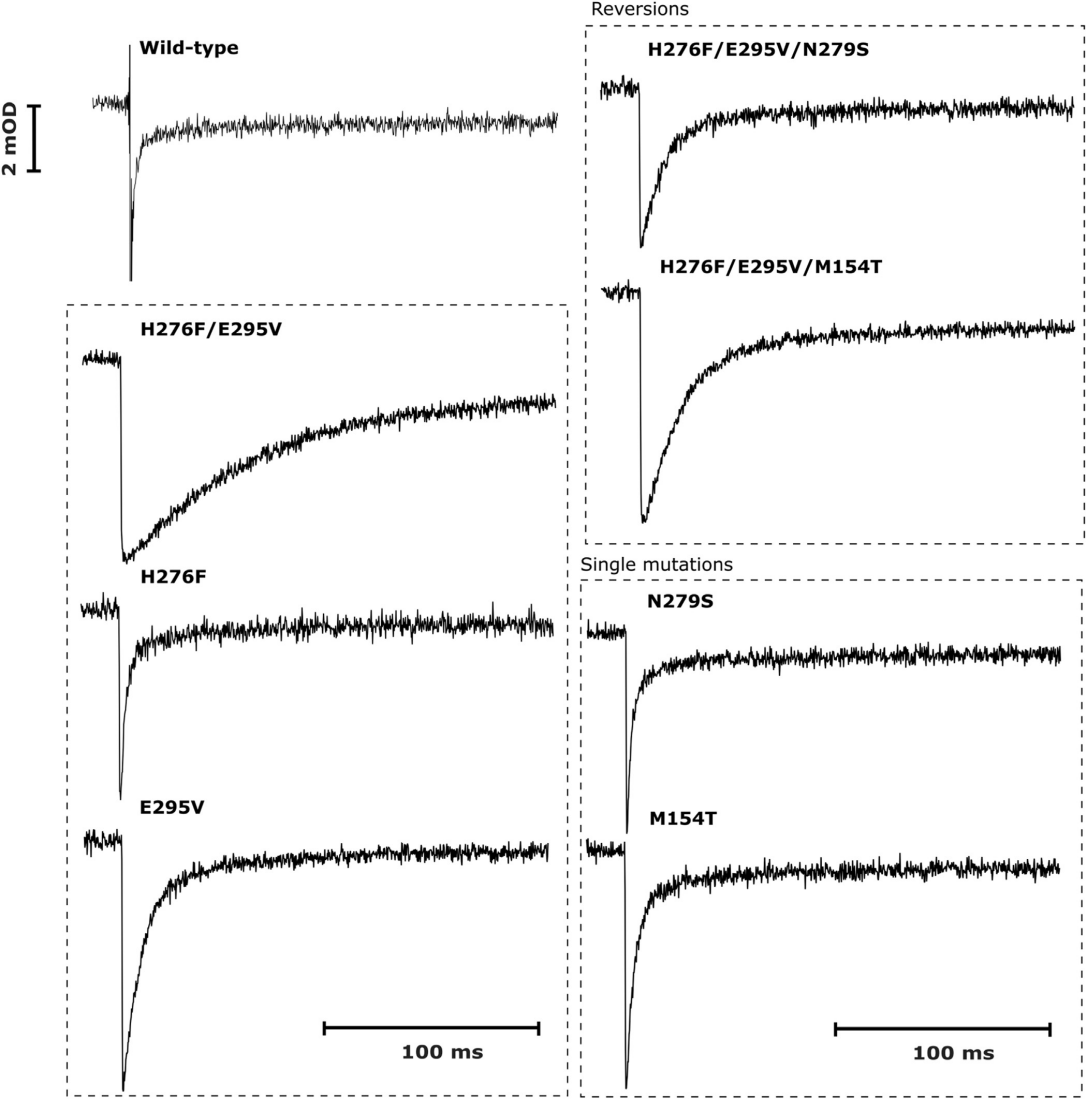
**Flash-induced cytochrome *c* oxidation and rereduction in chromatophores containing WT and mutated cytochrome *bc***_**1**_**.** Kinetic transients at 550 to 540 nm were recorded without inhibitors at pH 7 and ambient redox potential of 100 mV.

**Figure 4:**
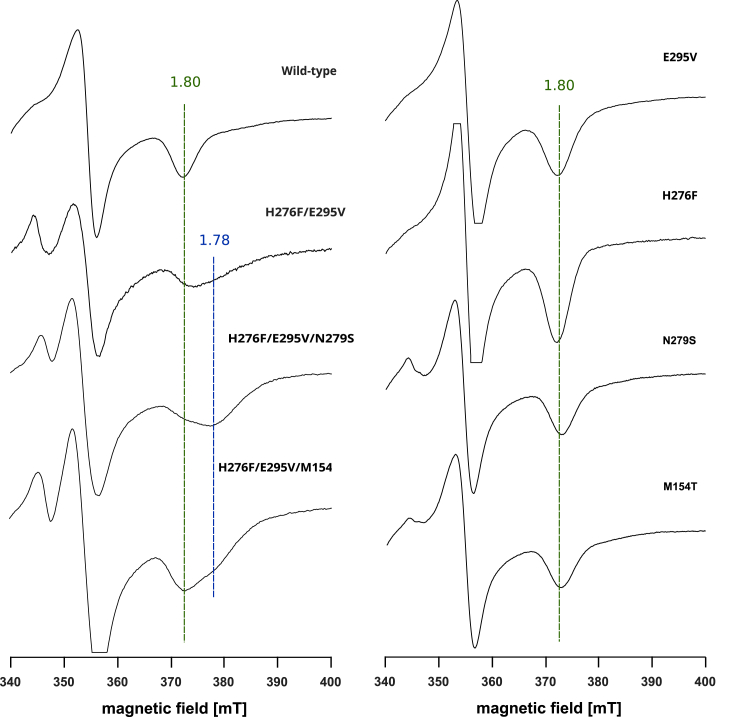
**CW EPR spectra of [2Fe–2S] cluster of WT and mutated cytochrome *bc***_**1**_**.** Chromatophore samples were reduced with sodium ascorbate. Spectra were recorded at 20 K. CW, continuous wave; EPR, electron paramagnetic resonance.

As shown in Figure 2*A*, the enzymatic activities of H276F were about 25% lower than those of the WT, at pH above 7. They, however, were still higher than activities of E295V, which showed significantly reduced activity at both low and high pH compared with WT. On the other hand, at pH below 7, enzymatic activity of H276F was comparable to the WT. This is due to the fact that histidine at pH below the p*K*a value preferentially exists in a protonated form and has little capacity for proton donation. It thus appears that under these conditions, it is irrelevant to the function of cytochrome *bc*_1_, whether there is a histidine or a nonprotonable amino acid at position 276. The rate of flash-induced cytochrome *c* reduction was decreased about 1.2 and 3.5 times for H276F and E295V, respectively, *versus* WT (Fig. 3, Table 1), which was consistent with the level of reduction in enzymatic activities discussed previously. We note that both single mutants, H276F and E295V, displayed native-like spectral properties of cofactors. The optical spectra of isolated complexes showed the presence of high-potential heme *c*_1_ and low potential hemes *b* (Fig. S2). Furthermore, the electron paramagnetic resonance (EPR) spectrum of reduced Rieske cluster displayed a characteristic g = 1.8 transition (Fig. 4), which reflects interactions of the cluster with quinone bound at the Q_o_ site. This confirmed the integrity of the catalytic Q_o_ site on both mutants and proper interaction of the subunits of the complex.

**Table 1:** Selected properties of various forms of cytochrome *bc*_1_

Bacterial strain	Photosynthetic growth^a^	EPR transition g_x_ of [2Fe–2S]	Flash-induced rate of cytochrome *c* reduction (s^−1^)
WT	Ps^+++^	1.8	380
H276F	Ps^+++^	1.8	315
E295V	Ps^+++^	1.8	110
H276F/E295V	Ps^–^	1.8 1.78	12
H276F/E295V/N279S	Ps^++^	1.8 1.78	87
H276F/E295V/M154T	Ps^+^	1.8 1.78	55
N279S	Ps^+++^	1.8	305
M154T	Ps^+++^	1.8	280

a Ps^−^ indicates photosynthetic incompetence; different growth rates under photosynthetic conditions are indicated by Ps^+^ to Ps^+++^ (from slow to native-like photosynthetic growth).

The observation that cytochrome *bc*_1_ remains functional in either of the single mutants (H276F or E295V) suggests that removal of just one protonable side chain in the region of the PEWY/*ef* loop is not enough to effectively switch off the proton transfer from the Q_o_ site. Similarly, the removal of any protonable side chain residue of the D252/K251 pair (D252A and K251M mutants) did not switch off the proton transfer to quinone at the Q_i_ site. This proton transfer was deactivated only in the double mutant (25). This prompted us to test the effect of simultaneous removal of both protonable side chains. Toward this end, we constructed and analyzed the H276F/E295V double mutant.

Similarly to the two single mutants, H276F/E295V displayed native-like optical spectral properties indicating the unchanged presence of heme components (Fig. S2). Interestingly, the EPR spectrum of the Rieske cluster showed a change in the shape of g_x_ transition, which became broader with the appearance of 1.78 component in addition to 1.8 characteristic for WT (Fig. 4). While these results confirmed the integrity of the Q_o_ site in H276F/E295V, they also pointed out toward some changes in the interaction of quinone with the Rieske cluster (see further discussion of this issue later).

Most importantly, in H276F/E295V, the functionality of the enzyme was lost as indicated by the inability of the cells to grow under photosynthetic conditions (Fig. S1, Table 1). Consistent with that observation, the enzymatic activity of H276F/E295V was lower than any of the single mutants over the entire pH range (Fig. 2*A*). Furthermore, the flash-induced rate of the cytochrome *c* reduction was decreased about 30 times *versus* WT (Fig. 3 and Table 1). Clearly, removal of both protonable residues had a synergistic effect in slowing down the enzymatic activity below the level required for generation of minimal proton motive force to enable the growth of the cells. We consider these results as an indication that the proton transfer is effectively switched off in H276F/E295V.

### Alternative solutions to restore proton transfer in inactive mutants

As the H276F/E295V strain was unable to grow photosynthetically (Ps^−^ phenotype), we screened this mutant for the photosynthetically competent (Ps^+^) revertant strains. Such analysis allowed us to identify two single mutations, M154T and N279S, in cytochrome *b* subunit (see Fig. 1, *B* and *D* for positions of reversions). Remarkably, each of them can individually compensate for the lack of both protonable residues in H276F/E295V without any other mutational changes (the original H276F and E295V are maintained). We thus obtained the triple mutants H276F/E295V/M154T and H276F/E295V/N279S. We note that both mutants exhibited slower, in comparison to WT, growth under photosynthetic conditions (Fig. S1).

Both reversions improved the enzymatic turnover, making the rates approximately two times larger comparing to H276F/E295V (Fig. 2*B*). They also resulted in increased rates of flash-induced cytochrome *c* reduction (Fig. 3). Triple mutants, as H276F/E295V, displayed unchanged optical spectral properties of hemes (Fig. S2) and EPR spectrum featuring g_x_ transition with 1.8 and 1.78 components (Fig. 4). We note that this effect was not observed in the single mutant M154T or N279S, constructed separately to examine if reversion mutations alone introduce significant changes to the protein complex. This clearly was not the case, as in addition to the native like-EPR spectrum of the Rieske cluster (with one g_x_ = 1.8 transition) (Fig. 4), M154T or N279S showed no change in the optical spectrum and relatively minor effect on the rate of flash-induced cytochrome *c* reduction (Fig. 3).

**Figure 5:**
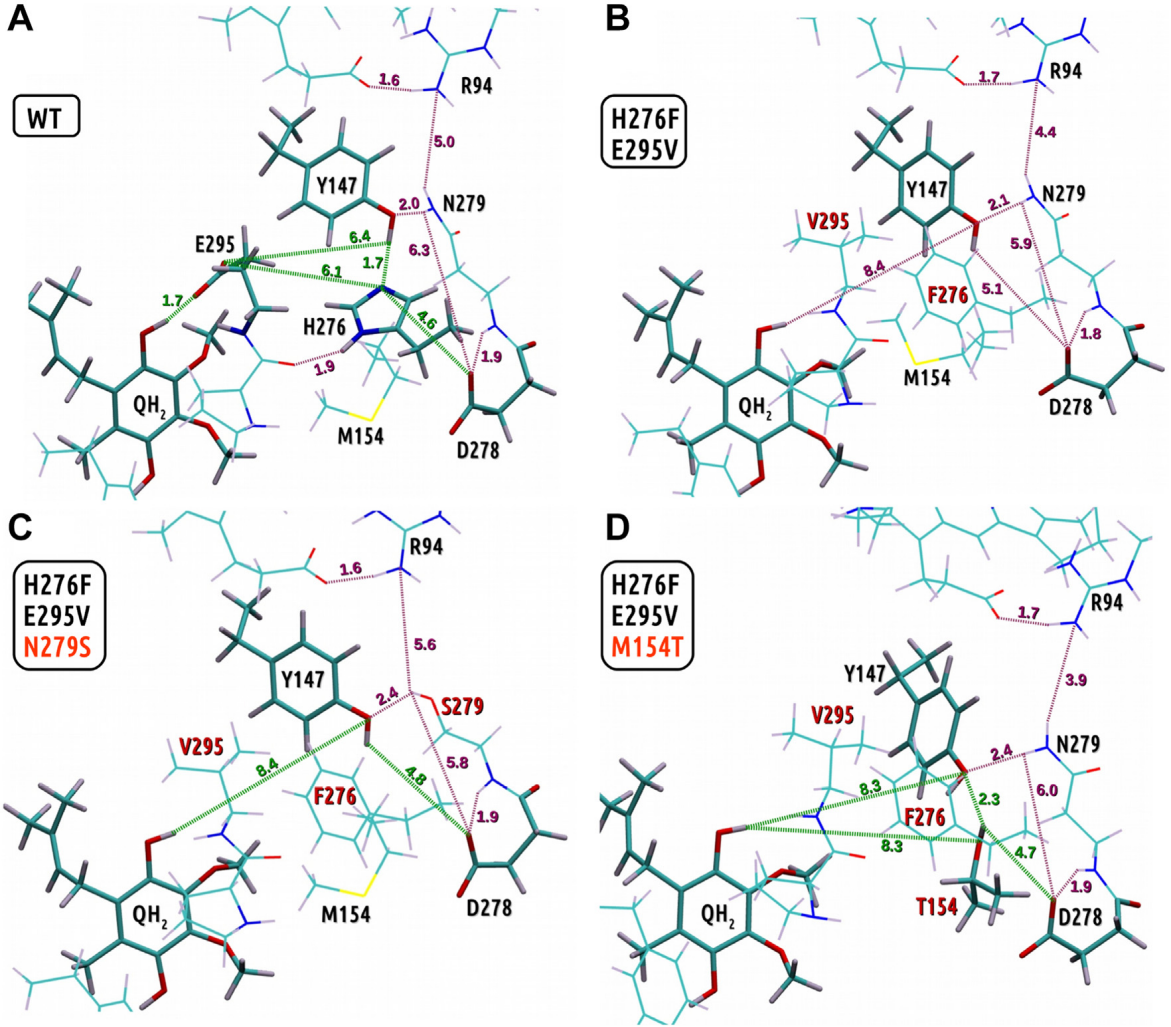
**Pattern of hydrogen bonds in the region of mutated residues in the optimized quantum mechanical models.***A,* WT, *B,* H276F/E295V mutant, *C*, H276F/E295V/N279S mutant, and *D,* H276F/E295V/M154T mutant. In *licorice* are presented residues that are presumably directly involved in the proton transfer. *Green dashed lines* show distances for proton moving from QH_2_ to D278, and *purple dashed lines* show other important distances. QH_2_, quinol.

**Figure 6:**
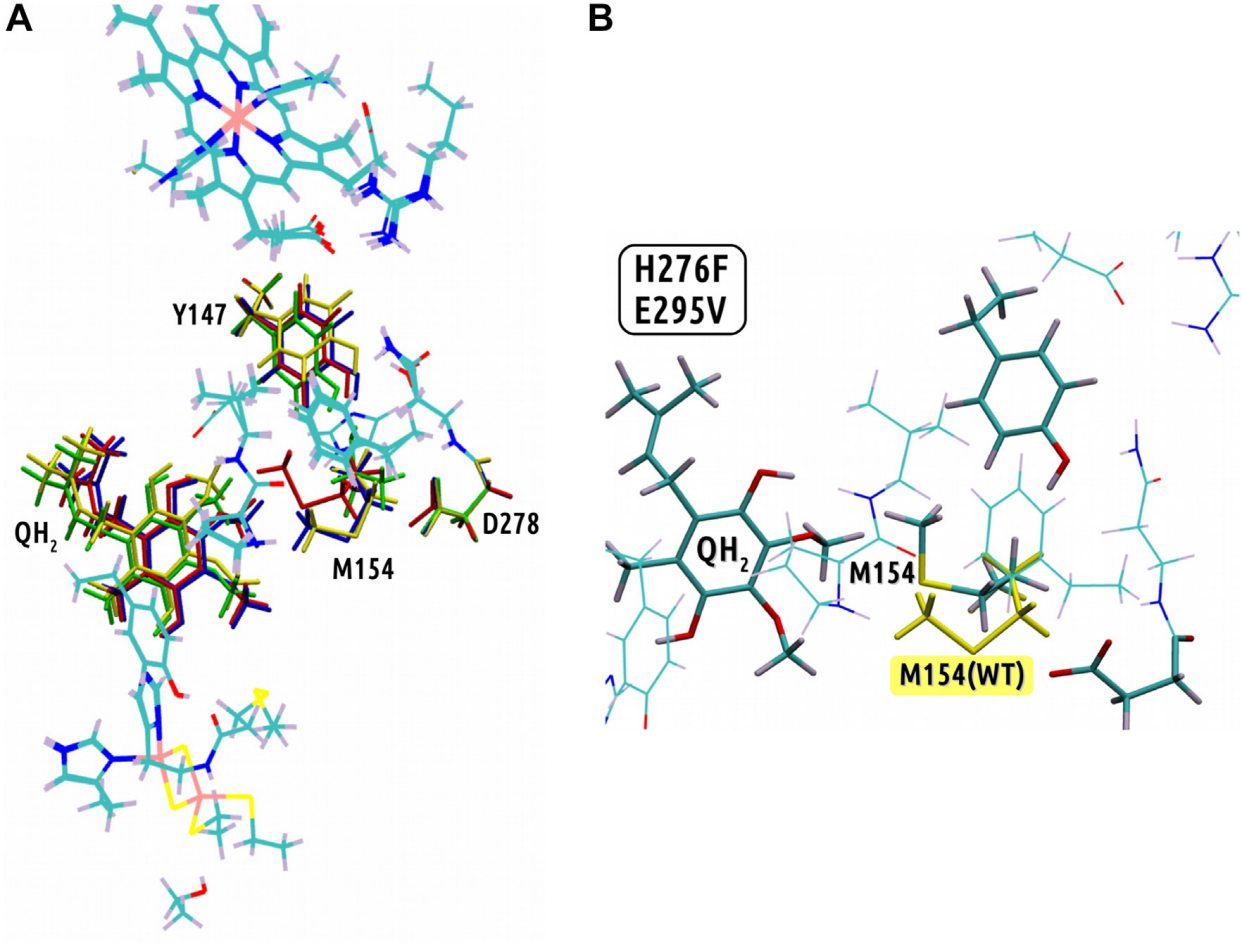
**Differences in the optimized structures of the considered variants of the Q**_**o**_**site.***A*, superimposed optimized geometries of quantum mechanical models constructed for WT and three mutants (H276F/E295V, H276F/E295V/M154T, and H276F/E295V/N279S). In *licorice*, representations show fragments of the structures, which revealed the largest conformational differences: *yellow*—WT, *red*—H276F/E295V, *green*—H276F/E295V/M154T, and *blue*—H276F/E295V/N279S. *B*, the conformation of M154 side chain observed in the optimized structure of H276F/E295V mutant, in reference to the conformation of M154 observed in WT and H276F/E295V/N279S mutant (added *yellow licorice*).

To get insights into the molecular basis of the restoration of the proton transfer competence in the triple mutants, we performed QM calculations, which included optimizations of geometries of the model of the active Q_o_ site for WT and three mutants: H276F/E295V, H276F/E295V/M154T, and H276F/E295V/N279S. The optimized structures with the substrate, QH_2_, bound at the site for all four cases are shown in Figures 5 and 6. QH_2_ was considered as the initial state in these calculations given that the sequence of electron and proton transfers leading to the oxidation of QH_2_ to Q at the Q_o_ site and the intermediate steps of this reaction remain uncertain.

The superimposition of all optimized structures revealed the largest conformational changes at position 147 and for the bound substrate (Fig. 6*A*). In addition, the structure of inactive mutant H276F/E295V showed large displacement of M154 side chain in comparison to other structures (*red licorice* in Fig. 6*A*). The detailed analysis of the hydrogen bond pattern for every model allowed us to consider various scenarios for proton transfer specific to a given case. This analysis focused on the transfer of proton occurring at the heme *b*_L_ side of the Q_o_ site, thus the region encompassing H276 and E295 (in native protein).

#### WT

The WT model (Fig. 5*A*) implicates that proton can be transferred from the substrate to E295 (1.7 Å) and then directly (6.1 Å) or *via* Y147 (6.4 Å) to H276 and D278 (4.6 Å). The short distances between the side chains of E295, Y147, and H276 make the proton transfer feasible even without involvement of water molecules. Moreover, Y147 creates strong hydrogen bonding interactions with H276 and amino group of N279 (2.0 Å), which in turn weakly interacts with R94 (5.0 Å). This pattern of hydrogen bonds constrains the conformation of Y147 in such a way that even though it cannot directly accept proton from the QH_2_, it can still assist in the proton transfer from E295 to H276.

#### H276F/E295V

In the absence of H276 and E295, Y147 emerges as the closest protonable residue at the heme *b*_L_ side of the Q_o_ site (Fig. 5*B*). However, the side chain of Y147 appears to be trapped at a position that is too distant from the bound QH_2_ to be an efficient proton acceptor. This could be caused by the loss of the hydrogen bond between Y147 and amino acid at position 276, which strengthens interaction between N279 and R94 (4.4 Å *versus* 5.0 Å) while maintaining the strong interaction between Y147 and N279 (2.1 Å). Interestingly, M154 significantly changes its conformation and interacts with hydrophobic residues V295 and F276 and aromatic ring of Y147. The shift in the position of the M154 is observed only in the structure of the H276F/E295V mutant (Fig. 6) and, in view of the structural effects of the reversions (see later and Fig. 5), is likely to be a crucial factor responsible for slowing down the catalytic rate. Indeed, the hydrophobic steric hindrance formed mainly by this residue with some contribution from V295 and F276 prevents proton uptake from QH_2_ by Y147. Additional steric hindrance caused by F276 may impede proton transfer from Y147 to D278 (5.1 Å).

#### H276F/E295V/N279S

Replacement of N279 by serine in H276F/E295V mutant weakens the hydrogen bond between Y147 and the residue at the 279 position (2.1 Å for N279 in H276F/E295V mutant *versus* 2.4 Å for S279 in H276F/E295V/N279S mutant) (Fig. 5*C*). This makes Y147 more mobile, releasing it from the fixed position in H276F/E295V, which in principle should help Y147 to accept the proton from the substrate. Moreover, serine is characterized by a shorter side chain than asparagine, thus in the H276F/E295V/N279S mutant, Y147 is situated closer to D278 (proton acceptor) than in the H276F/E295V mutant (4.8 Å *versus* 5.1 Å). Also, the interaction between the residues at positions 279 and R94 is significantly weakened (4.4 Å for N279 in H276F/E295V mutant *versus* 5.6 Å for S279 in H276F/E295V/N279S), which should help S279 to assist in proton transfer from Y147 to D278. Importantly, the side chain of M154 switches back to the position observed in WT, where it interacts with the aromatic ring of QH_2_. M154 in the WT conformation no longer provides steric hindrance for Y147 to approach the substrate and uptake proton from it.

#### H276F/E295V/M154T

A replacement of M154 by shorter hydrophilic residue threonine emerges as an alternative way to remove the steric hindrance for Y147 introduced by a specific conformation of M154 in H276F/E295V mutant (Fig. 5*D*). T154 forms a hydrogen bond with Y147, which weakens the hydrogen bond between Y147 and N279 (from 2.1 Å in H276F/E295V to 2.4 Å in H276F/E295V/M154T) and enables Y147 to approach toward the QH_2_. The Y147–T154 and T154–D278 distances equal 2.3 Å and 4.7 Å, respectively, thus T154 can, in principle, mediate proton transfer from Y147 to D278.

Given the observed differences in the shape of EPR spectra of the double and triple mutants (Fig. 4), we also compared interactions of the Rieske cluster with QH_2_ in the various QM models (Fig. S3). Such analysis revealed that shortening of the distance between the His156 and hydroxyl group of the QH_2_ by about 0.2 to 0.3 Å (1.8 or 1.7 Å *versus* 2 Å in WT) could be associated with the broader EPR g_x_ transition in those mutants. Of note, binding of stigmatellin, a potent inhibitor of the Q_o_ site, also results in shortening of the distance between His156 and the quinone (7), which appears to be reflected in the change of g_x_ transition from 1.8 to 1.77 (26). However, stigmatellin still forms a hydrogen bond with E295, which is not the case in the double mutants or triple mutants. Perhaps any, even subtle, changes in the orientation (and distance) between histidine and the quinone/QH_2_ cause changes in the shape of this transition, making it a very sensitive spectroscopic probe. The exact origin of g_x_ transition is still not certain and beyond the scope of this article. Nevertheless, the EPR spectra of Figure 4 indicate that alterations of the interactions with QH_2_ at the heme *b*_L_ side (in our case, the effect of the simultaneous presence of H276F and E295V) might induce some changes in the interaction of QH_2_ with the Rieske cluster.

### Extended hydrogen bonding network for proton transfer exposed by mutations

The results identify specific conformational configurations at the heme *b*_L_ side of the Q_o_ site that can effectively modulate proton transfer associated with the catalytic reaction. In the native protein, two protonable residues (H276 and E295) line up in the direction from the bound QH_2_ toward D278 and in a configuration that secures highly efficient proton transfer. The two single mutants (H276F or E295V) still operate at functional, yet reduced compared with WT, catalytic rates, and only when both mutations are introduced (H276F/E295V), the functional activity of the enzyme is lost. This indicates that at the minimum, one protonable residue must be placed in position facilitating proton transfer from the bound QH_2_ in a direction toward D278.

The H276F/E295V mutant contains Y147—a protonable residue that could still potentially engage in proton transfer toward D278 (Fig. 5*B*). However, Y147 appears to be stacked between N279, M154, V295, and F276, in a position too far from the QH_2_ for efficient uptake of proton. Such a trapped position of Y147, which is separated from QH_2_ by hydrophobic residues, not only prevents Y147 from directly accepting a proton from QH_2_ but also from the participation of water molecules in the proton transfer. The structural effects of the reversions N279S or M154T reveal two conformational features that allow the H276F/E295V mutant to restore the enzymatic activity. First, the side chain of Y147 needs to gain mobility by weakening the interaction between Y147 and the residue at position 279 and removing the steric hindrance introduced by M154. Restoring the position of M154 observed in the WT form of the enzyme will not only allow Y147 to approach QH_2_ but also create space for water molecules that can mediate the proton uptake. Second, the interaction between Y147 and D278 needs to be improved, which can be achieved by shortening the distance between Y147 and D278 or introducing another hydrophilic residue that facilitates the proton transfer between Y147 and D278.

Both configurations engage Y147. We thus propose that in the absence of protonable residues in positions 276 and 295 (H276F/E295V), Y147 contributes a major role in proton transfer from QH_2_ to D278. We further argue that the specific effects of the mutants discussed previously exposed Y147 as a residue that is likely to contribute to the network for proton transfer also in the native protein. Indeed, such a role for Y147 has already been considered and discussed in the literature (14, 27, 28).

Earlier work on Y147 demonstrated that selected mutants of this residue (namely Y147S and Y147A) were not functional *in vivo* but regained functionality when additional mutation M154V was present (27). It is intriguing that M154V concerns the amino acid position at which one of our reversions was identified (M154T). Thus, to understand these results in the context of our work, we performed QM calculations of two additional configurations: the Y147S single mutant and the Y147S/M154V double mutant.

**Figure 7:**
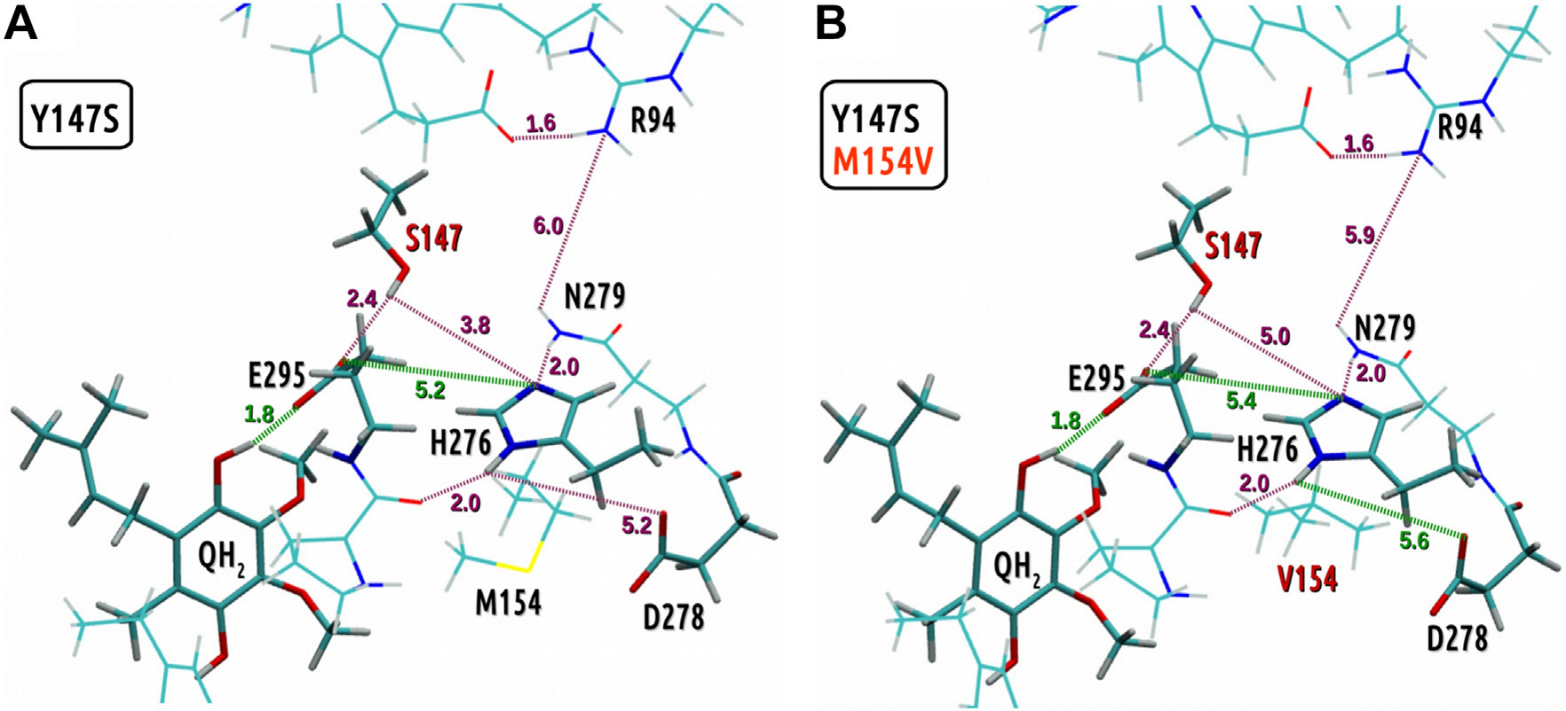
**Pattern of hydrogen bonds in the region of substituted residues in the optimized quantum mechanical models.**  *A*, Y147S mutant, *B,* Y147S/M154V mutant. Color code for the *dashed lines* is as in Figure 2.

**Figure 8:**
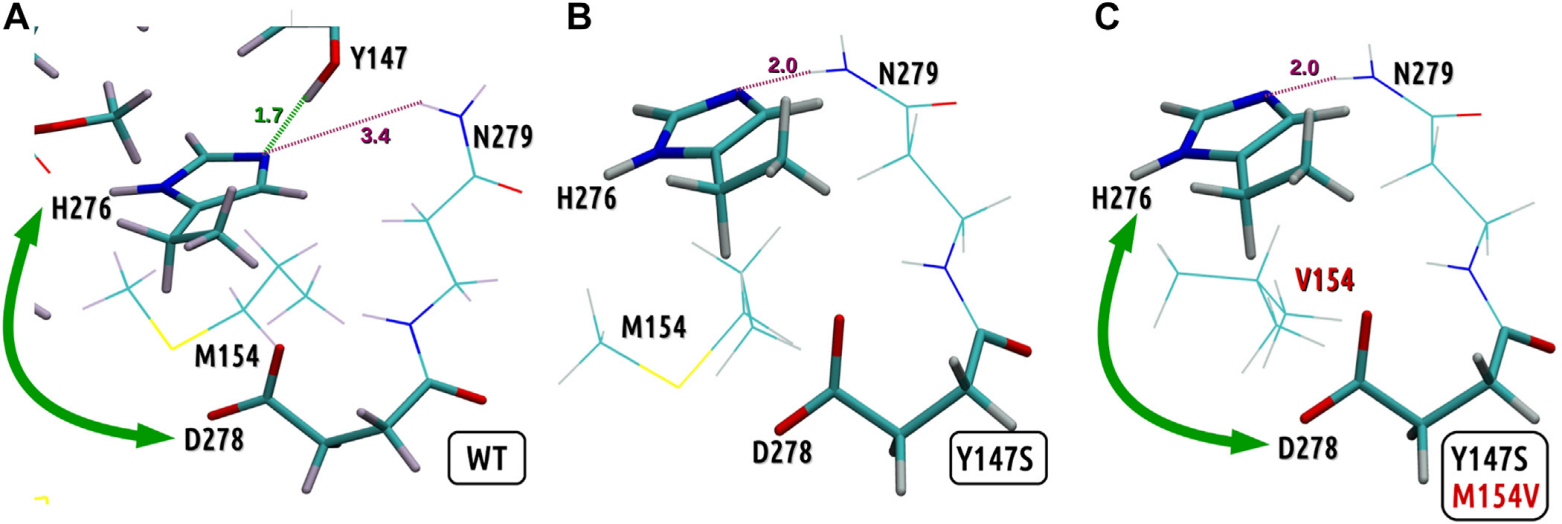
**Fragments of optimized geometries of quantum mechanical models representing the relative conformations of N279, H276, M154(V154), and D278.***A,* WT, *B,* Y147S mutant, and *C,* Y147S/M154V mutant. The *green arrow* shows the possibility of conformational change of H276 to transfer a proton to D278.

The results revealed that replacement of Y147 with serine resulted in a loss of hydrogen bond between this position and H276 (serine is significantly smaller than tyrosine, thus its hydroxyl group is positioned too far from H276 side chain; Fig. 7*A*). The lost hydrogen bond was compensated by a strong hydrogen bond between N279 and H276 (2.0 Å with respect to 3.4 Å in WT; Fig. 8). As a result, the position of H276 changed causing M154 to become a steric hindrance for the delivery of proton toward D278. Of note, a similarly small alanine is expected to exert a similar structural effect, explaining the nonfunctionality of the Y147A mutant. In the Y147S/M154V, the strong hydrogen bond between N279 and H276 was maintained, but replacing M154 with a smaller residue removed the steric hindrance in this region. Consequently, the side chain of H276 gained mobility, which overall facilitated proton transfer toward D278.

It thus appears that the steric hindrance provided by M154 under an altered hydrogen bond pattern is the common effect of all inactive mutants (H276F/E295V and Y147S or Y147A). This hindrance causes a decrease in the mobility of protonable Y147 or H276 and blocks the influx of water molecules, which effectively inhibits the proton transfer.

## Conclusions

**Figure 9:**
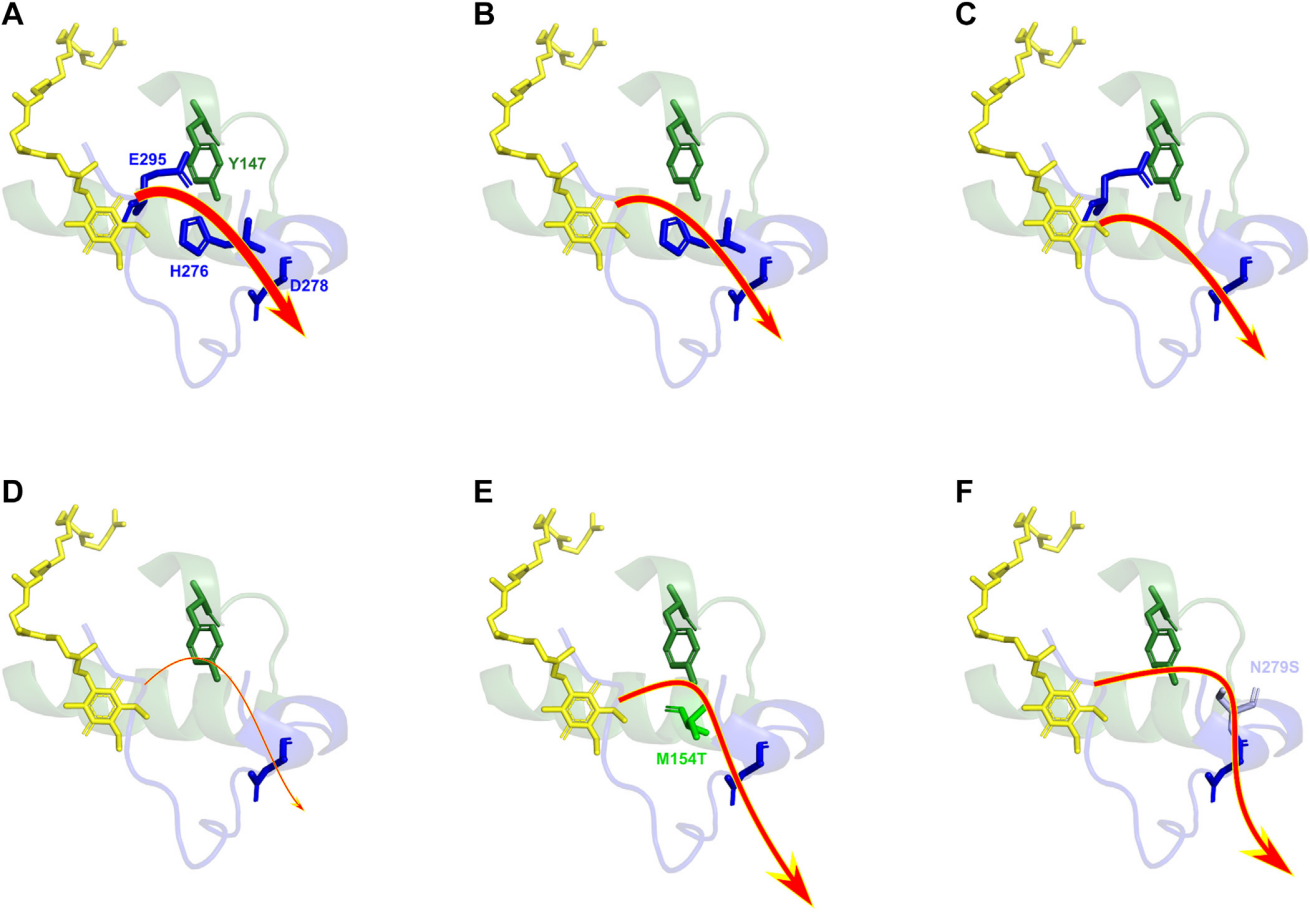
**Schematic illustration of proton transfer pathways in theconsideredforms of cytochrome *bc***_**1**_**.***A*, WT, *B*, E295V, *C*, H276F, *D*, H276F/E295V, *E*, H276F/E295V/M154T, and *F*, H276F/E295V/N279S. The thickness of the *red arrow* reflects various levels of the overall efficiency of proton transfer: the thicker the line, the higher probability of proton transfer. *Yellow sticks* and *light-blue ribbon* represent the ubiquinone molecule and *ef*-loop, respectively.

Figure 9 schematically summarizes our main conclusions about the efficiency of proton transfer at the heme *b*_L_ side of the Q_o_ site in the native enzyme and various mutants studied here.

We observed that cytochrome *bc*_1_ functionality is lost by introducing a double mutation H276F/E295V, which severely impedes the proton transfer (Fig. 9*D*). Introduction of a single mutation H276F or E295V lowers the catalytic capabilities of the enzyme but maintains its functionality at a sufficient level (Fig. 9, *B* and *C*). The geometries of optimized QM models indicate that residues at positions 276 and 295 are at the hydrogen-bonding distance from Y147. Replacing both H276 and E295 with nonprotonable residues results in a significant conformational change of Y147. The altered conformation of M154 provides a steric hindrance for Y147 to approach the QH_2_ and limits the available space preventing the participation of water molecules in proton transport. Consequently, the connection of Y147 with QH_2_ is lost (Figs. 5*B* and 6).

This connection is restored in the H276F/E295V mutant when additional mutations N279S or M154T are present (Fig. 9, *E* and *F*). In the triple mutants H276F/E295V/N279S or H276F/E295V/M154T, the residue 154 does not provide steric hindrance anymore, and Y147 adopts a conformation similar to that observed in the WT (Fig. 6).

Earlier studies revealed that Y147S mutation causes a loss of the enzyme activity with a compensatory effect provided by mutation M154V (27). Our QM calculations imply that in the absence of Y147, its role can be taken over by H276. This, however, requires that the side chain at position 154 does not provide steric hindrance, which is secured by the M154V mutation.

In summary, our study exposed dynamic operation of an extended hydrogen bonding network, build up by E295, H276, D278, and Y147. Various alterations of this network by point mutations exert a more or less severe inhibitory effect on the proton transfer. General flexibility of the entire network and its surroundings secures some level of tolerance to mutational change without overall loss of function. In such cases, conformation mobility of specific residues that stay in play appears critical to maintain hydrogen bond connectivity for efficient proton transfer from the Q_o_ site.

## Experimental procedures

### Preparation of mutants

The mutant E295V was from the study by Osyczka *et al.* (19). To construct other mutants of *R. capsulatus* examined in this work, a genetic system originally developed by Dr F. Daldal (University of Pennsylvania, Philadelphia, PA) (29) was used. First, mutations were introduced to the appropriate pPET plasmid. To construct the single mutation H276F, the pPET plasmid containing WT gene coding for cytochrome *b* (petB) was used as a template in QuikChange site-directed mutagenesis method (Agilent Technologies) with the following PCR primers (mutated nucleotides in underlined triplets are marked in *bold*):

H276F_Forward: 5'–CTACCTCGGC**TT**CCCGGACAAC–3';

H276F_Reverse: 5'–GTTGTCCGGG**AA**GCCGAGGTAG–3'.

Single mutants M154T and N279S were constructed in a similar way, using the following primers:

M154T_Forward: 5'–GCCGTGGGGCCAGA**C**GTCGTTCTGGGGCG–3';

M154T_Reverse: 5'–CGCCCCAGAACGAC**G**TCTGGCCCCACGGC–3';

N279S_Forward: 5'–GGCCACCCGGACA**G**CTACGTCCAGGCC–3';

N279S_Reverse: 5'–GGCCTGGACGTAG**C**TGTCCGGGTGGCC–3'.

The double mutation H276F/E295V was constructed by introducing a single H276F mutation to the pPET plasmid bearing E295V mutation in the *petB* gene using the same method and primers for H276F. Next, the XmaI/AsuII (or BstXI/XmaI in case of M154T) fragment of the operon containing the desired mutations and no other mutations was exchanged with WT counterpart of pMTS1 expression plasmid to construct pMTS1:bH276F, pMTS1:bH276F/E295V, pMTS1:bM154T, and pMTS1:bN279S. The resulting pMTS1 plasmids were introduced into MT-RBC1 *R. capsulatus* strain (devoid of *petABC* operon) using triparental crossing. The presence of introduced mutations was confirmed by sequencing of the exchanged DNA fragments on plasmids isolated from the mutated *R. capsulatus* strains.

### Photosynthetic selection of revertants

*R. capsulatus* bacteria were grown under semiaerobic or photoheterotrophic conditions as described previously (30). To select the reversions, the photosynthetically inactive (Ps^−^ phenotype) mutant strain H276F/E295V was grown on MPYE plates under photosynthetic/anaerobic conditions for 10 days (31). Single colonies that regained photosynthetic competence (Ps^+^ phenotype) were isolated and subjected to further analysis. The DNA sequencing of the entire *petABC* operon of the plasmid isolated from Ps^+^ single colonies revealed the presence of original mutations (H276F/E295V) and the appearance of additional substitutions (second-site reversions) in cytochrome *b* sequence: methionine to threonine at position 154 (three of six analyzed colonies) or asparagine to serine at position 279 (three of six analyzed colonies). No other mutations were detected in the entire *petABC* operon. These strains were thus named H276F/E295V/M154T or H276F/E295V/N279S, respectively.

The test for bacterial ability to grow under the photosynthetic conditions was performed by diluting 2 ml overnight liquid cultures of each mutant to an absorbance of 0.1 at 600 nm in 200 μl MPYE and reduction plating on the appropriate section of MPYE-Kan agar plates with bacteriological loop. The plate was incubated under photosynthetic/anaerobic conditions for 7 days, photographed daily. A copy of the plate was also incubated under aerobic/dark conditions as a control.

### Steady-state kinetics measurements

Enzymatic activity of the isolated cytochrome *bc*_1_ complexes was determined spectroscopically by 2,3-dimethoxy-5-methyl-6-decyl-1,4-benzohydroquinone-dependent reduction of cytochrome *c* (bovine heart cytochrome *c* from Sigma–Aldrich) as described previously (29). All enzymatic assays were performed in 50 mM MES, MOPS, or Tris buffer (for pH ranges: 5.5–6.0, 6.5–7.5, and 8.0–8.5, respectively) containing 0.01% *n*-dodecyl β-d-maltoside without salt ions. The low ionic strength was to ensure high efficiency of electron transfer from cytochrome *c*_1_ to cytochrome *c*, which in turn allowed the observation of the limitations of reaction at the level of QH_2_ oxidation at the Q_o_ site. The final substrate concentrations were 20 μM 2,3-dimethoxy-5-methyl-6-decyl-1,4-benzohydroquinone and 20 μM cytochrome *c*. Concentrations of cytochrome *bc*_1_ were in the range of 10 to 100 nM, depending on the activity of the mutant. Turnover rates were calculated from the initial linear parts of kinetic traces. For all forms of enzymes, the pH dependence of the steady-state enzymatic activity was conducted. This dependence can be simulated well by the equation:

(Eq. 1) $$v=\frac{V_{prot}+V_{deprot}\times 10^{\text{pH}-\text{pKa}}}{1+10^{\text{pH}-\text{pKa}}}$$

where *K*_a_ is the acid dissociation constant, *V*_*prot*_ and *V*_*deprot*_ are turnover rates of enzymes in protonated (low pH value) and deprotonated (high pH value) states, respectively. This equation is based on the assumption that enzymatic activity depends on the protonation state of the amino acids in the vicinity of the Q_o_ site. The enzyme in the protonated form (at low pH) has the lowest activity, the enzyme in the deprotonated form has the highest activity (at high pH), whereas the observed enzymatic activity at intermediate pH values is a mix of these both.

### QM calculations

Six large cluster QM models of Q_o_ active site (349–364 atoms) were constructed based on the Protein Data Bank 1ZRT crystal structure solved for *R. capsulatus* cytochrome *bc*_1_ in complex with stigmatellin (32). The cluster models were built for the Q_o_ site in complex with ubiquinol (QH_2_), which was inserted in the position of stigmatellin. The cluster models were constructed for WT enzyme and five mutants: H276F/E295V, H276F/E295V/M154T, H276F/E295V/N279S, Y147S, and Y147S/M154V. Each QM model consisted of oxidized heme *b*_L_, oxidized [2Fe–2S], QH_2_, and 18 protein residues: R94, H97, H198, Y147(S147), M154(T154, V154), H276(F276), D278, N279(S279), P294, E295(V295), and Y302 from chain P, and C133, H135, C138, C153, C155, H156, and S158 from chain E. The following residues were considered with their Cα carbons, and the NH and CO moieties from protein backbone were replaced with hydrogens: R94, H97, H198, Y147(S147), M154(T154, V154), H276(F276), Y302, C133, H135, C138, C153, and S158. In case of dipeptide fragments: D278–N279(S279), P294–E295(V295), and C155–H156, the peptide bond was included in the model, and only NH group of D278 and C155 and CO group from N279(S279), E295(V295), and H156 were replaced by hydrogen atoms.

The geometry of Q_o_–QH_2_ complexes was optimized using DFT/B3LYP method in combination with Grimme's D3 dispersion correction computed with Becke–Johnson dumping (33, 34) and def2-SVP double dzeta basis set. Geometry optimization was performed using Gaussian 16 program (35). During geometry optimization, constraints were imposed on the carbon and hydrogen atoms of the protein backbone in order to preserve the rigidity of the protein backbone.

### Flash-induced redox kinetics of hemes

Double-wavelength time-resolved spectrophotometer was used to measure the kinetics of electron transfer through the heme cofactors of cytochrome *bc*_1_. Measurements were performed using chromatophores suspended in buffer: 50 mM MOPS; pH 7.0; 100 mM KCl; and 1 mM EDTA. Samples were poised at ambient redox potential of 100 mV under anaerobic conditions in the presence of 3.5 μM valinomycin and the following redox mediators: 7 μM 2,3,5,6-tetramethyl-1,4-phenylenediamine, 1 μM phenazine methosulfate, 1 μM phenazine ethosulfate, 5.5 μM 1,2-naphthoquinone, and 5.5 μM 2-hydroxy-1,4-naphthoquinone, according to the previously described method (29). Transient kinetics of c-type heme oxidation and rereduction were monitored at 550 to 540 nm after activation by single saturating light pulse (∼10 μs). The rates of flash-induced electron transfer reactions were determined from single exponential function fitted to heme *c* rereduction without the presence of any inhibitors.

### EPR spectroscopy

Continuous wave EPR spectra of [2Fe–2S] clusters were measured at X band frequency (9.4 GHz) using membrane chromatophores. Samples of WT and mutants were prepared in buffer: 50 mM Mops; pH 7.0; 100 mM KCl; 1 mM EDTA. Samples were reduced with sodium ascorbate and quickly frozen in liquid nitrogen. The EPR measurements were carried at 20 K on a Bruker Elexsys E580 spectrometer using a SHQEOS11 resonator combined with ESR900 Oxford Instruments cryostat and the Stinger cryocooler (ColdEdge). The parameters are the following: resonance frequency, 9.78 GHz; microwave power, 1.9 mW; and modulation amplitude, 10 G.

## Data availability

The data that support the findings of this study are available from the corresponding author, A.O., upon request.

## Supporting information

This article contains supporting information.

## Conflict of interest

The authors declare that they have no conflicts of interest with the contents of this article.
