# Supplementary material for: Identification of hydrogen bonding network for proton transfer at the quinol oxidation site of Rhodobacter capsulatus cytochrome bc1
Source: J Biol Chem. 2023 Sep 14;299(10):105249. doi: 10.1016/j.jbc.2023.105249 (PMC10583091; doi:10.1016/j.jbc.2023.105249)
Supplement: Supporting Figures S1–S3 [file mmc1.pdf]

Supporting Information

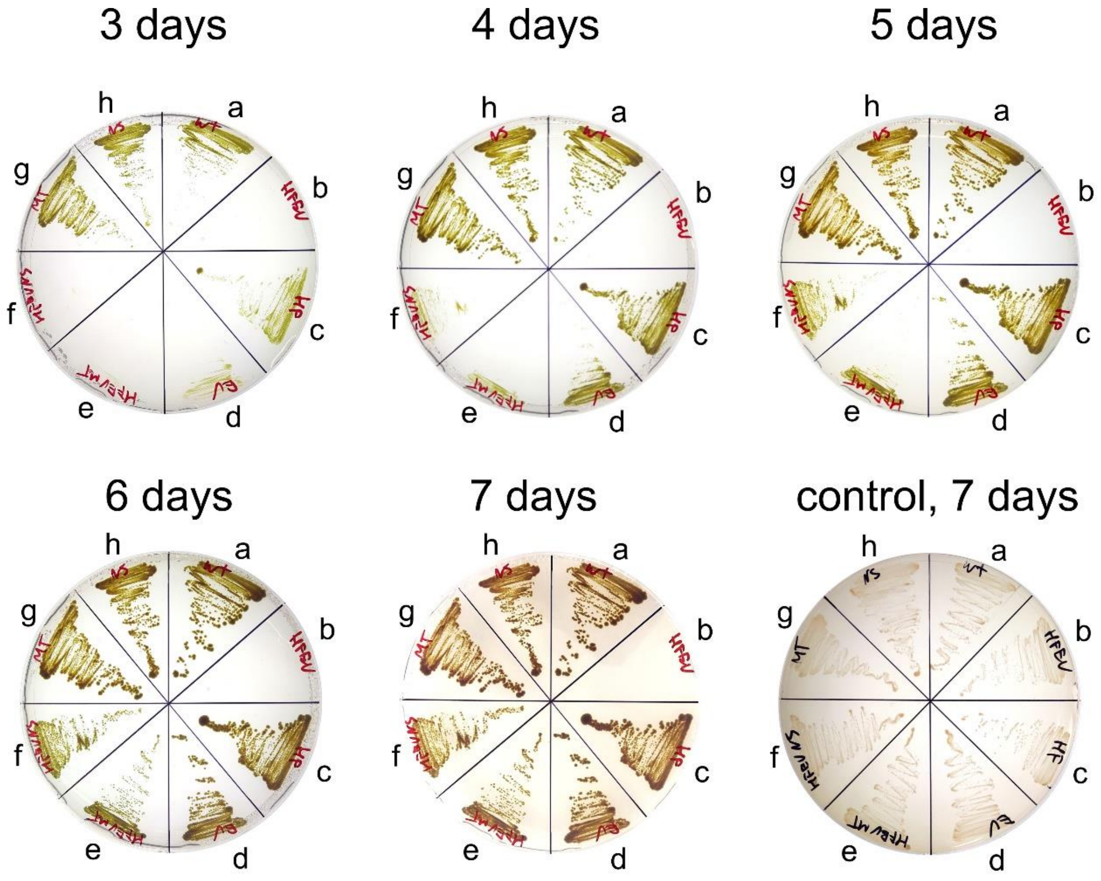

Figure S1. Photosynthetic growth of various *R. capsulatus* strains documented daily after seeding the bacteria (3 – 7 days). The letters *a*, *b*, *c*, *d*, *e*, *f*, *g* and *h* indicate WT, H276F/E295V, H276F, E295V, H276F/E295V/M154T, H276F/E295V/N279S, M154T and N279S respectively. Control plate was cultured under aerobic/dark conditions for 7 days.

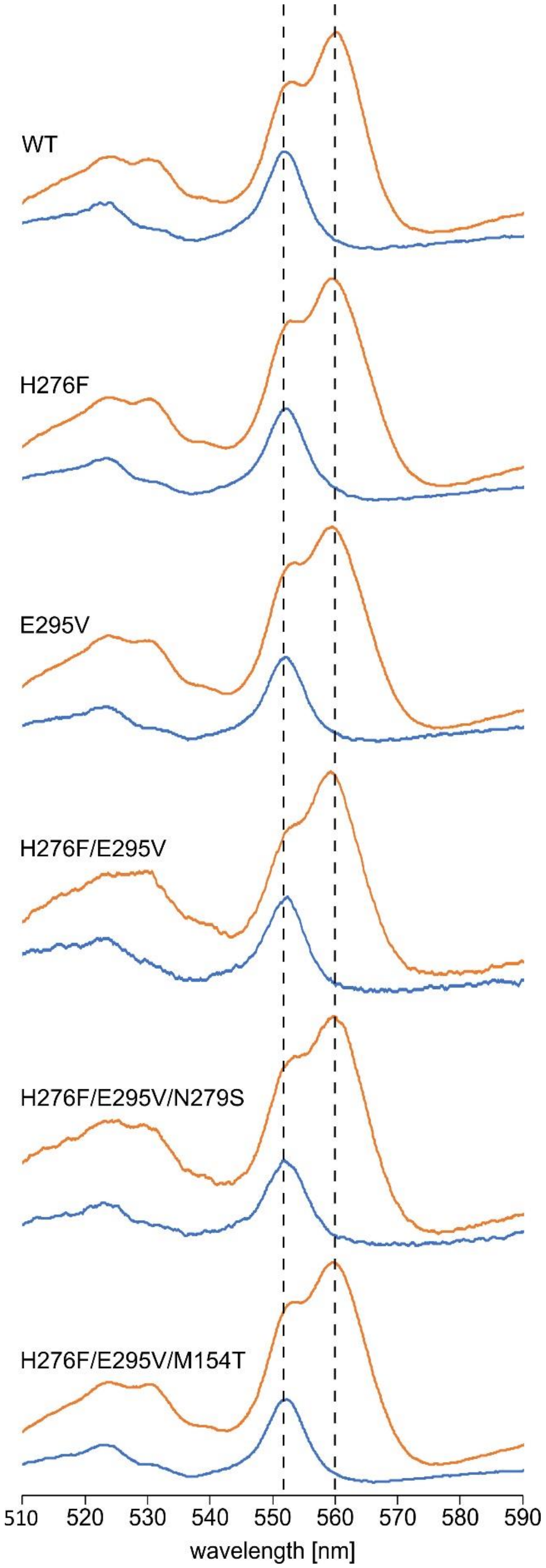

Figure S2. Optical difference spectra of purified cytochrome *bc*$_1$ complexes. Blue and orange lines correspond to ascorbate minus ferricyanide and dithionite minus ferricyanide spectra, respectively. Dotted lines indicate the maxima of absorption for heme c_1 (552 nm) and hemes *b* (560 nm).

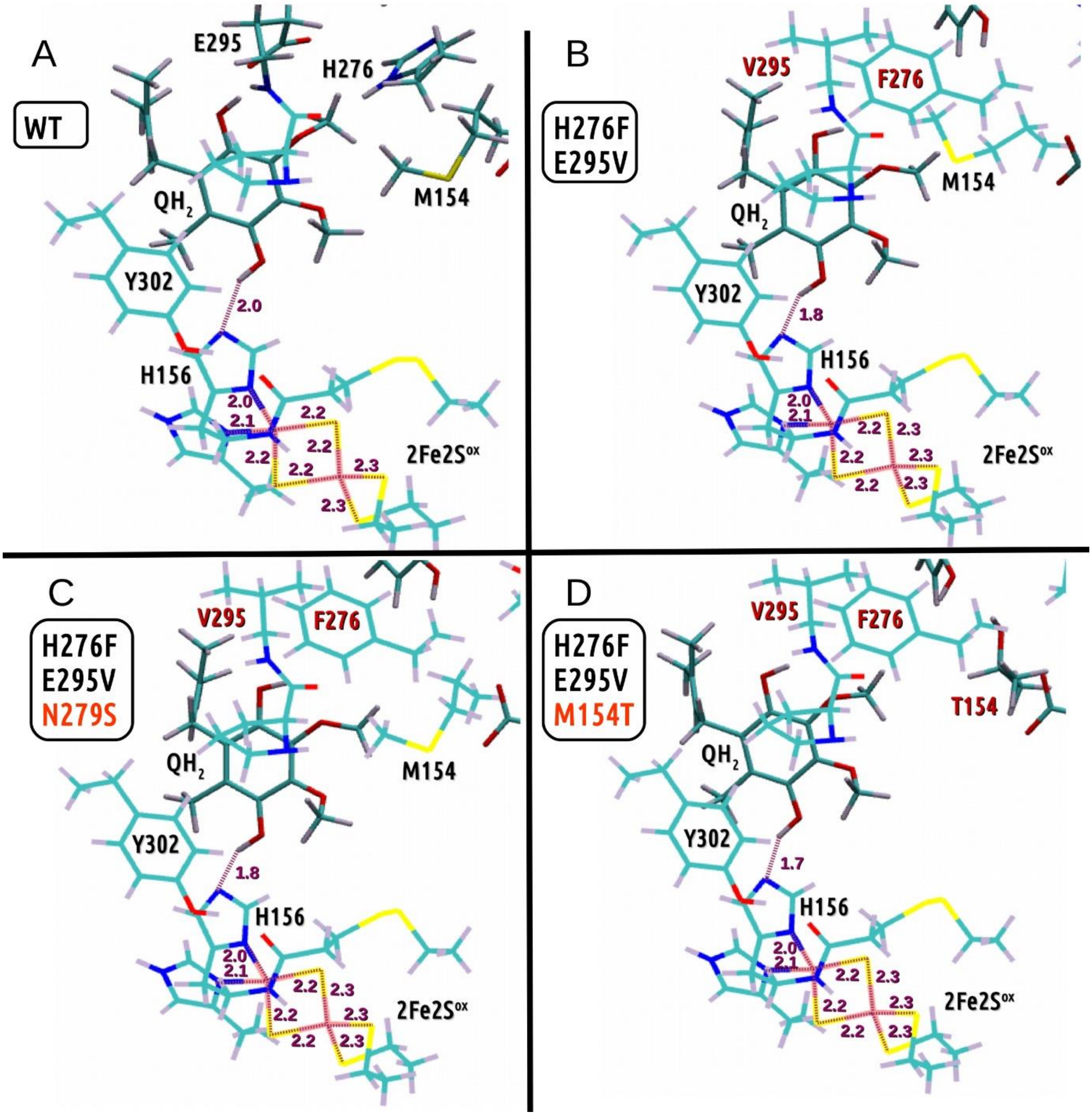

Figure S3. Interaction between quinol and oxidized [2Fe-2S] cluster in the optimized quantum mechanical models. *A*, wild type, *B*, H276F/E295V mutant, *C*, H276F/E295V/N279S mutant, and *D*, H276F/E295V/M154T mutant. Marked distances are in Ångstroms.
